# Judo specific fitness test performance variation from morning to evening: specific warm-ups impacts performance and its diurnal amplitude in female judokas

**DOI:** 10.1186/s13102-022-00484-4

**Published:** 2022-05-21

**Authors:** Özgür Eken, Filipe Manuel Clemente, Hadi Nobari

**Affiliations:** 1 Physical Education and Sports Teaching, Faculty of Sport Science, Inonu University, Battalgazi, Malatya, Turkey; 2 Escola Superior Desporto e Lazer, Instituto Politécnico de Viana do Castelo, Rua Escola Industrial e Comercial de Nun’Álvares, 4900-347 Viana do Castelo, Portugal; 3 Research Center in Sports Performance, Recreation, Innovation and Technology (SPRINT), 4960-320 Melgaço, Portugal; 4 Delegação da Covilhã, Instituto de Telecomunicações, 1049-001 Lisbon, Portugal; 5 Department of Exercise Physiology, Faculty of Educational Sciences and Psychology, University of Mohaghegh Ardabili, Ardabil, 5619911367 Iran; 6 Faculty of Sport Sciences, University of Extremadura, 10003 Cáceres, Spain; 7 Department of Motor Performance, Faculty of Physical Education and Mountain Sports, Transilvania University of Braşov, 500068 Braşov, Romania

**Keywords:** Combat sports, Diurnal variation, Martial arts, Specific testing, RAMP protocol

## Abstract

**Background:**

A number of specific tests are used to standardize competition performance. Specific Judo fitness test (SJFT) can be applied by considering the start of the competition qualifiers in the morning and the continuation of the final competitions in the evening. The improvement of test performances can be achieved with warm-up for elevating heart rate (HR) and muscle temperature such as raise, activate, mobilise, potentiate (RAMP) protocols.

**Purpose:**

The aim of this study is to evaluate the effects of different warm-up protocols on SJFT at different times of the day in female judokas.

**Methods:**

Ten volunteer women participated in this study, who regularly participated in judo training for more than 5 years and actively competed in international competitions. Judokas completed SJFT, either after no warm-up, or RAMP protocols like specific warm-up (SWU), and dynamic warm-up for two times a day in the morning: 09:00–10:00 and in the evening: 16:00–17:00, with at least 2 days between test sessions. The following variables were recorded: throws performed during series A, B, and C; the total number of throws; HR immediately and 1 min after the test, and test index after different warm-ups.

**Results:**

When analyzed evening compared to the morning without discriminating three warm-up protocols, evening results statistically significant number of total throws performed during series A, B, and C, the total number of throws; HR immediately and 1 min after the test, and test index than morning results (*p* < 0.01). Moreover, RAMP protocols interaction with time have demonstrated an impact on SJFT for index [F_(2)_ = 4.15, *p* = 0.024, η_p_^2^: 0.19] and changes after 1 min HR [F_(1.370)=_ 7.16, *p* = 0.008, η_p_^2^: 0.29]. HR after 1 min and test index results were statistically significant in favor of SWU (*p* < 0.05).

**Conclusions:**

In conclusion, SJFT performance showed diurnal variation and judo performances of the judokas can be affected more positively in the evening hours especially after RAMP protocols.

**Supplementary Information:**

The online version contains supplementary material available at 10.1186/s13102-022-00484-4.

## Background

Judo can be understood as an acyclic sport in which ultimate performance is constrained by the dynamic of the combat and in which biological mechanisms (like somatic maturity or biological age) [1, 2], technical/tactical skills [3–5], psychological conditions and environment (as time of the day) play an important role [6, 7]. Taking into consideration that a combat may range 4– min (regular combat or extended to golden score), the energetic profile of judo is mixed depending on both anaerobic and aerobic metabolisms [8], in which aerobic power and neuromuscular power can sustain the range, frequency and intensity of actions and movements that judokas need to perform to defeat the opponent [6, 9–14].

Since the combat is short term, prompt physical and physiological readiness is required of judokas. Since judokas require neuromuscular readiness for combat, one of the strategies to implement for starting at maximal level to adequately warm up is required for preparedness for judo match at the competition [15]. Different approaches could be done before judo competition or match. Some approaches may use post activation potentiation to induce neural readiness for the immediate power movements, which appears to be effective [16]. Other way is to apply high load inspiratory muscle warm-up, although with absence of effectiveness in simulated judo tests [17]. Another way is to apply dynamic stretching promoting mobility, with possible effects on leg strength [18]. Other hypotheses are the conventional jogging and/or running and combat-based exercises [16]. When some research in the literature are examined, sauna sessions are stated to be used as a potential warm-up [19, 20]. Besides raise, activate, mobilize, and empower (RAMP) the most appropriate opportunity to address critical performance components such as speed, agility, and skill practice, including a highly targeted, progressive phase in skill development and a progressive intensity structure for judo activities, can be beneficial. The RAMP structure addresses previous shortcomings and enables the planning and execution of targeted actions throughout the warm-up sequence. RAMP’s effect on performance improvements prior to the specific Judo fitness test (SJFT) is an exciting topic [21–23].

Being complex to determine the effects of different warm-up protocols on official judo combats, some alternatives can be using some tests with the proximity of judo combat which can provide some references for researchers. One of the well-accepted tests to simulate judo demands is the SJFT [24]. This test comprises three periods of judo activity (e.g., Period A: 15 s; Periods B and C: 30 s), interspaced by 10 s of rest. The test allows judokas to perform *ippon-seoinage* technique [24]. The heart rate (HR) is collected immediately after the test and one minute after to determine HR recovery profile. The test is highly influenced by anaerobic metabolism (possibly as consequence of high-intensity and repeated efforts) but also for aerobic metabolism, thus possibly simulating the effects of a judo combat on judokas [25, 26].

Using SJFT as a reference test, a study compared different warm-up protocols (based on post activation potentiation vs. conventional) revealing the beneficial effects of post activation potentiation on performance and peak power [16]. However, is still not possible to reveal the different effects that a multitude of warm-up protocols can induce in performance during SJFT. Moreover, effects of warm-up can be constrained by the time of the day. Naturally, the time of the day also plays an important role since is linked with circadian rhythm and the biological and hormonal responses to this rhythm. As an example, judoka’s muscle power and strength seems to be significantly higher in the afternoon than in the morning [27]. Naturally, sleep quality and athletic performance may act as mediators or moderators for those variations, however it seems that the period of the day is critical for ultimate physical and physiological performance in judo and other sports [28].

More research is need about the effects of different warm-up protocols on judokas performance, while considering time of the day as an important factor to identify such an effect. Research that provides such a design may help coaches to identify the most adequate scenario and warm-up to positively influence the readiness of judokas for combat. Besides, the improvement of test performances can be achieved with a warm-up for elevating HR and muscle temperature such as RAMP protocols. Therefore, the aim of this study was two-fold: (1) analyze the effects of different warm-up protocols on the performance of judokas determined during SJFT; and (2) identify the possible interactions of warm-ups with time of the day.

## Method and material

### Participants

Ten volunteer women (mean age: 19.10 ± 1.16 years, mean height: 161.10 ± 3.90 cm, mean body mass: 59.20 ± 8.66 kg, body mass index: 22.70 ± 2.44 kg/m^2^, HR_rest_: 61.60 ± 5.25 bpm), who regularly participated in judo training for more than 5 years, and actively competed in international competitions, participated in this study. While five of the judokas competed actively in international competitions, the remaining five finished in the top three at national competitions. All athletes were proficient in the ippon-seoi-nage throwing technique and had been engaging in resistance training twice a week for at least a year. Study was conducted 3 weeks after the national tournament. The air temperature during the warm-up and fitness tests were 26–28 °C (using the Kestrel 4500 Pocket Weather Tracker, Nielsen- Kellerman Co., USA). Before starting the study, the volunteers were given detailed information about the content, purpose and methodological model of the study. Informed consent form was signed by the subjects who stated that they volunteered to participate in the study. In addition, the study was carried out according to international ethical standards for human biological rhythm research [29]. Prior to the study, participants were asked to sleep for at least 8 h before each testing session. In addition, they were asked to come full, provided that they ate at least two hours before the morning and evening sessions (Additional file 1). All test and assesments applied in this study were approved by the Institute’s Clinical Research Ethics Committee (Approval Number: 2021/2520). Additionally, participants were informed about the importance of refraining from high-intensity exercise and the avoidance of substances such as alcohol and caffeine during the implementation and testing phases of the protocols [30].

### Procedures

The SJFT performance of the participants was assessed after different warm-up protocols including; no warm-up (NWU), specific warm-up (SWU), and dynamic warm-up (DWU) in two different time periods of the day (morning: 09:00–10:00 h, and evening: 16:00–17:00 h) with at least two days between each other [27]. Also, the reason why these time periods of the day were chosen for the study was related to the fact that judo competitions follow a course from morning (eliminations) to evening (finals). The study consisted of 3 warm up protocols as NWU (only 30–40% of HR_max_, 15 min. jogging), DWU (30–40% of HR_max_, 5 min jogging + 10 min. dynamic warm up exercise), and SWU (30–40% of HR_max_, 5 min jogging + 10 min. judo-specific warm up). The Karvonen formula was used to calculate HR reserves of the judo athletes before each test sessions [31]. Polar H10 was used to monitor HR during 5 min of jogging, and after SJFT performance. All of the protocols consist of 15 min. This study continued approximately 12 days. All protocols continued for consecutive days.

### Warm-up protocols

*No warm up (NWU)* The warm up rate was determined according to the 30–40% HR_max_ of each subject [31]. Subjects were light jogging for only 15 min under the control of the experts. In this way, both warm up intensity and warm up differences between participants in the training were eliminated. After 15 min’ light jogging, subjects’ SJFT were performed.

*Specific warm up (SWU)* Subjects were light jogging for only 5 min under the control of the experts according to the 30–40% HR_max_ of each subject [31]. After light jogging, SWU exercise was made. This warm up consisted of 10 SWU (foot sweeps, finger wrist and ankle rotations, trunk side stretch, trunk rotator stretch, hip circles, knee bends, cartwheels both sides, forwards rolls, backwards rolls, and forward rolls with legs spread) exercises (Table 1) [32]. They performed all SWU exercise totally 10 min.

**Table 1:** Specific warm-up (SWU) protocol

SWU protocol	Description
Foot sweeps	Moving side to side, sweep foot along floor
Finger, wrist, and ankle rotations	Rotate ankles and wrists to stretch flexors and extensors
Trunk side stretch	Standing, lean to one side then the other with arms overhead
Trunk rotator stretch	Standing, rotate body from side to side
Hip circles	On all fours, circle hip inside body and away from body, switch
Knee bends	Bouncing from kneeling position to standing position, stretch hamstrings
Cartwheels both sides	Standing facing the side, cartwheel
Forwards rolls	Standing, perform a forward roll into side body landing
Backwards rolls	Standing, perform a backward roll
Forward rolls with legs spread	Same forward roll with spread legs

*Dynamic warm up (DWU)* Subjects were light jogging for only 5 min under the control of the experts according to the 30–40% maxHR of each subject [31]. After 5 min jogging, judokas performed DWU exercise. This stretching exercise consisted of 10 DWU exercises that improved from moderate to high intensity (high knee pulls, straight-leg march, power skip, light skip, high glute pulls, light high knees, light butt kicks, rapid high knees, carioca, and walking lunge) (Table 2) [33]. They performed all DWU exercise totally 10 min.

**Table 2:** Dynamic warm-up (DWU) protocol

DWU protocol	Description
High-knee walk	While walking, lift knee towards chest, raise body on toes, and swing alternating arms
Straight-leg march	While walking with both arms extended in front of body, lift one extended leg towards hands then return to starting position before repeating with other leg
Hand walk	With hands and feet on the ground and limbs extended, walk feet towards hands while keeping legs extended then walk hands forward while keeping limbs extended
Lunge walks	Lunge forward with alternating legs while keeping torso vertical
Backward lunge	Move backwards by reaching each leg as far back as possible
High-knee skip	While skipping, emphasize height, high- knee lift, and arm action
Lateral shuffle	Move laterally quickly without crossing feet
Back pedal	While keeping feet under hips, take small steps to move backwards rapidly
Heel-ups	Rapidly kick heels towards buttocks while moving forward
High-knee run	Emphasize knee lift and arm swing while moving forward quickly

### Study variables

Body weights were measured with an electronic scale (Tanita SC-330S, Amsterdam, Netherlands) with an accuracy of 0.1 kg. During the measurement, the height of the participants was measured with a stadiometer (Seca Ltd., Bonn, Germany) with precision of 0.01 m (m). Body mass index and body fat ratios of all volunteers were measured and recorded with an electronic scale (Tanita SC-330S, Amsterdam, Netherlands) [34].

### Special judo fitness test

**Figure. 1:**
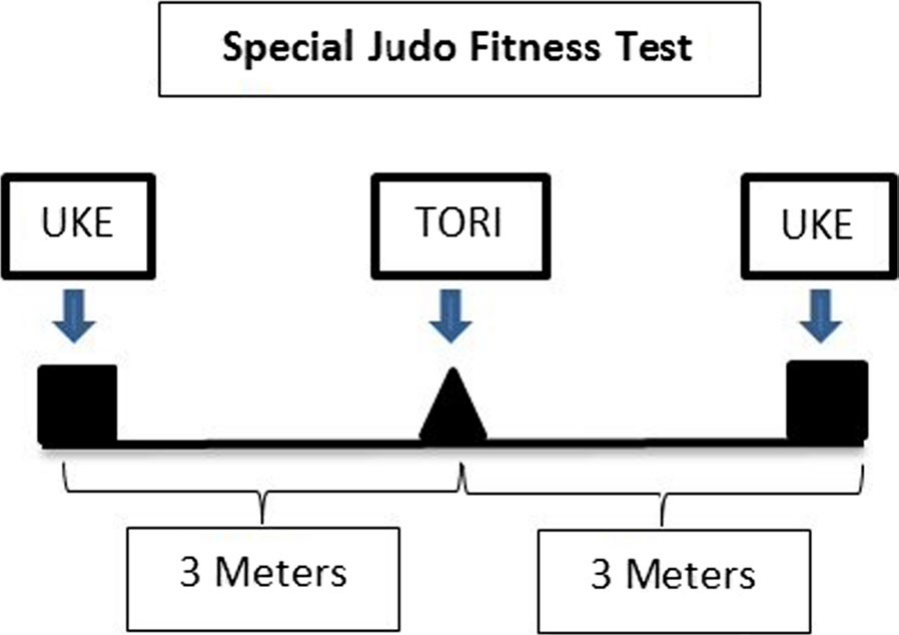
Design of specific Judo fitness test; TORI: The judoka who practices the technique; UKE: The judoka to whom the technique is applied

This SJFT was developed by Sterkowicz and was previously described by Franchini et al. [35, 36]. Three athletes of similar body mass are needed to perform the SJFT: 1 participant (TORI: The judoka who practices the technique) is evaluated, and 2 other individuals receive throws (UKE: The judoka to whom the technique is applied). The tori begins the test between the 2 ukes (3 m away from each uke). On a signal, the tori runs to one of the ukes and employs a throwing technique called ippon-seoi-nage. The tori then immediately runs to the other uke and completes another throw. The athlete must complete as many throws as possible within the test time. The SJFT is composed of three parts (15 (series A), 30 (series B), and 30 (series C) seconds) separated by 10 s recovery periods. The total number of throws completed by the tori during each of the three periods was recorded; the tori's heart rate (HR) was measured immediately after and 1 min after the test (Polar Team 2, Polar, Finland). The SJFT index was calculated according to the following equation: Index = (HR after + HR 1 min after)/total number of throws. The index value decreases with better test performance. Reliability values for this test were reported as 0.97 [37]. Figure 1 shows the SJFT [38].

### Statistical analysis

The statistical analysis was initially carried out using the ‘Shapiro Wilks’ normality test and the homoscedasticity test. All the variables presented normal distribution and homoscedasticity. Two-way repeated-measures ANOVA was used to assess differences in SJFT performance (total series of seri A, B, C, total scores, index, HR after warm up protocols and 1 min after warm up protocols) according to different warm-up protocols (NW, DWU, and SWU) in two different times of day (morning and evening). The sphericity was checked using ‘Mauchly’s Test’. When the assumption of sphericity was not met, the significance of the F ratios was adjusted according to the ‘Greenhouse–Geisser’ procedure. Pairwise tests were run to further investigate the effect of each condition. To determine the significance of significant findings, statistical effect sizes were calculated using partial eta-square (η_p_^2^) [39]. The effect sizes were calculated and classified to determine the magnitude of changes among the experimental conditions as proposed by ‘Cohen’s d’. An effect size classified as 0.2 was deemed small, 0.5 medium, and 0.8 large [40]. The findings are presented as mean ± standard deviation (SD). An alpha level of *p* < 0.05 was considered statistically significant for all analyses. All data analysis was conducted using SPSS version 25.0 (SPSS, Inc., Chicago, IL, USA).

## Results

**Fig. 2:**
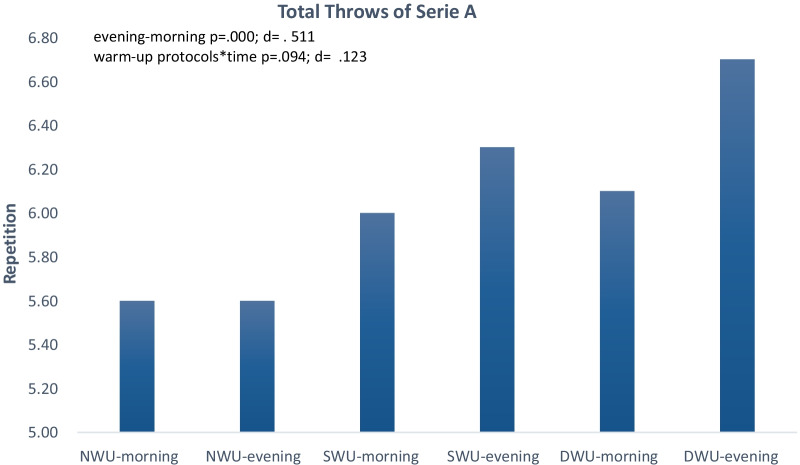
Mean and SD of SJFT for series A in the morning and evening hours of three warm-up protocols

In Fig. 2, there was a significant increase in series A, evening compared to the morning without discriminating three warm-up protocols (F_(2)=_18.84 *p* ≤ 0.001, η_p_^2^: 0.51). Moreover, warm-up protocols × time (evening and morning) interaction did not significantly have an impact on SJFT for series A (F_(2)=_2.53, *p* = 0.094, η_p_^2^: 0.12). When analyzed series A values within themselves, it was determined that NWU morning 5.60 ± 0.516, evening 5.60 ± 0.516; SWU morning 6.00 ± ≤ 0.001 evening 6.30 ± 0.483; DWU morning 6.10 ± 0.316 evening 6.70 ± 0.674.

**Fig. 3:**
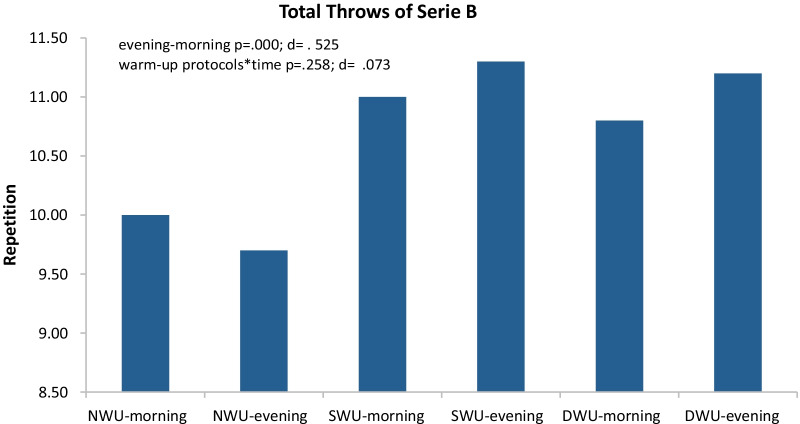
Mean and SD of SJFT for series B in the morning and evening hours of three warm-up protocols

In Fig. 3, there was a significant increase in series B, evening compared to the morning without discriminating three warm-up protocols (F_(2)=_19.87 *p* ≤ 0.001, η_p_^2^: 0.53). Moreover, warm-up protocols × time (evening and morning) interaction did not significantly have an impact on SJFT for series B (F_(2)=_1.41, *p* = 0.258, η_p_^2^: 0.07). When analyzed series B values within themselves, it was determined that NWU morning 10.00 ±  ≤ 0.001, evening 9.70 ± 0.948; SWU morning 11.00 ± 0.816 evening 11.30 ± 1.059; DWU morning 10.80 ± 0.918 evening 11.20 ± 1.135.

**Fig. 4:**
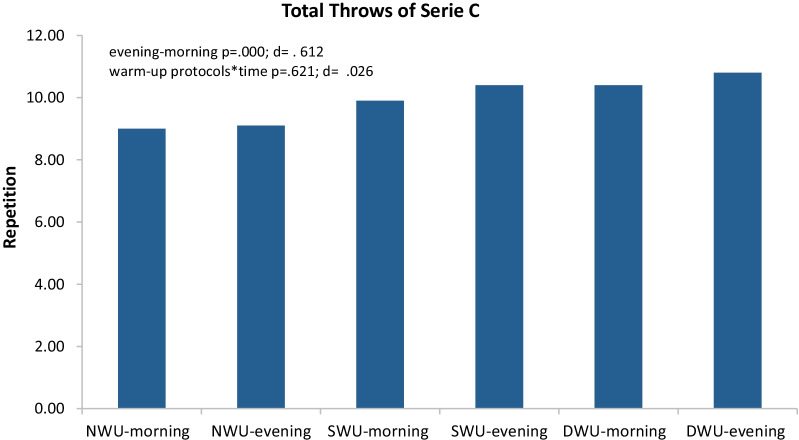
Mean and SD of SJFT for series C in the morning and evening hours of three warm-up protocols

In Fig. 4, there was a significant increase in series C, evening compared to the morning without discriminating three warm-up protocols (F_(2)=_12.72 *p* ≤ 0.001, η_p_^2^: 0.61). Moreover, warm-up protocols × time (evening and morning) interaction did not significantly have an impact on SJFT for series C (F_(2)=_ 0.48, *p* = 0.621, η_p_^2^: 0.03). When analyzed series C values within themselves, it was determined that NWU morning 9.00 ± 0.471, evening 9.10 ± 0.737; SWU morning 9.90 ± 0.316 evening 10.40 ± 0.699; DWU morning 10.40 ± 0.966 evening 10.80 ± 0.632.

**Fig. 5:**
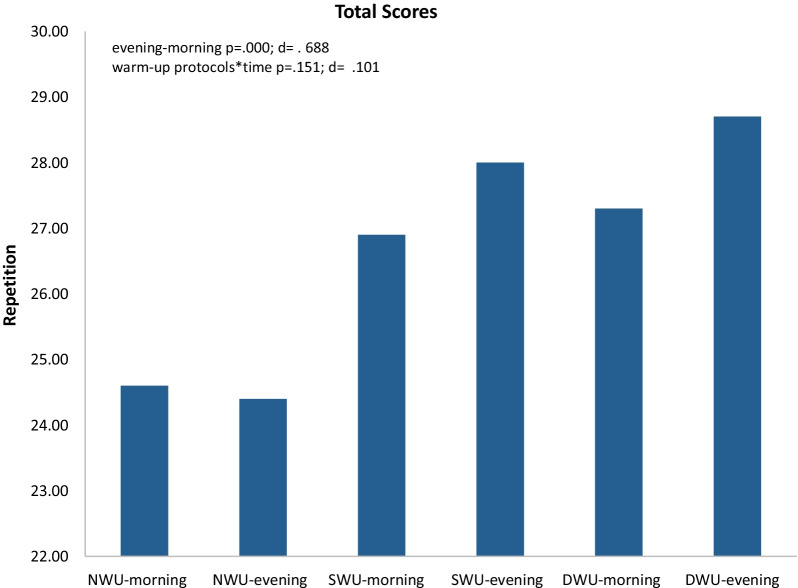
Mean and SD of SJFT for total scores in the morning and evening hours of three warm-up protocols

In Fig. 5, there was a significant increase in total scores, evening compared to the morning without discriminating three warm-up protocols (F_(1.853)=_39.61 *p* ≤ 0.001, η_p_^2^: 0.69). Moreover, warm-up protocols × time (evening and morning) interaction did not significantly have an impact on SJFT for series C (F_(1.853)=_ 2.02, *p* = 0.151, η_p_^2^: 0.10). When analyzed series total scores within themselves, it was determined that NWU morning 24.60 ± 0.843, evening 24.40 ± 1.712; SWU morning 26.90 ± 0.994 evening 28.00 ± 1.247; DWU morning 27.30 ± 1.766 evening 28.70 ± 1.946.

**Fig. 6:**
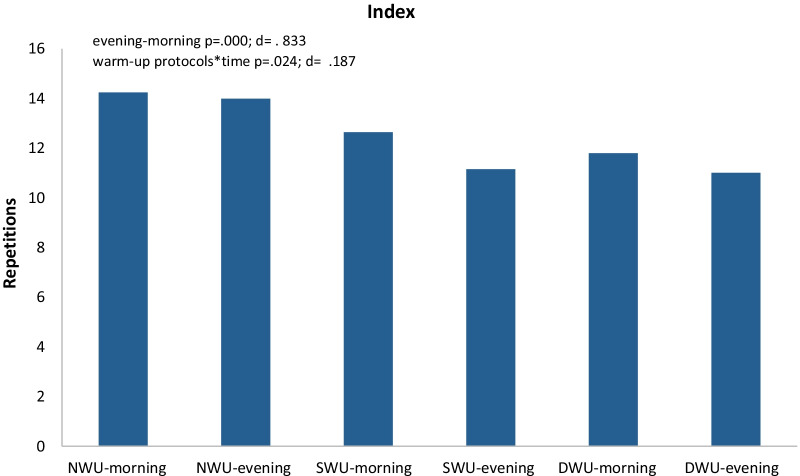
Mean and SD of SJFT for index in the morning and evening hours of three warm-up protocols

In Fig. 6, there was a significant increase in index, evening compared to the morning without discriminating three warm-up protocols (F_(2)=_89.92 *p* ≤ 0.001, η_p_^2^: 0.83). Moreover, warm-up protocols × time (evening and morning) interaction have an impact on SJFT for index (F_(2)=_ 4.15, *p* = 0.024, η_p_^2^: 0.19). When the significant differences between the groups are examined, there are significant differences between NW and SWU (*p* ≤ 0.001), NW and DWU (*p* ≤ 0.001) and SWU and DWU (*p* < 0.027) values. When analyzed index values within themselves, it was determined that NWU morning 14.24 ± 0.487, evening 13.98 ± 1.391; SWU morning 12.64 ± 0.474 evening 11.15 ± 0.557; DWU morning 11.79 ± 0.697 evening 11.00 ± 0.684. Results further showed that the DWU had the lowest probability of index according to all other protocols (*p* < 0.05).

**Fig. 7:**
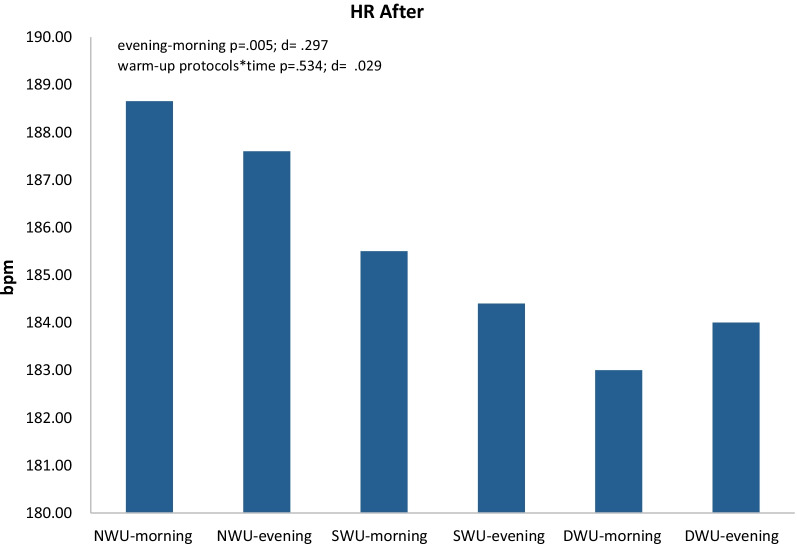
Changes in heart rate (HR) after different three warm-up protocols [no warm-up (NWU), specific warm-up (SWU), and dynamic warm-up (DWU)] in the morning and evening hours

In Fig. 7, there was a significant increase in HR after, evening compared to the morning without discriminating three warm-up protocols (F_(1.44)=_7.61 *p* = 0.005, η_p_^2^: 0.30). Moreover, warm-up protocols × time (evening and morning) interaction did not significantly have an impact on SJFT for HR after (F_(1.44)=_ 0.54, *p* = 0.534, η_p_^2^: 0.03). When analyzed changes in heart rate values within themselves, it was determined that NWU morning 189.00 ± 4.082, evening 187.60 ± 5.125; SWU morning 185.50 ± 7.153 evening 184.40 ± 5.966; DWU morning 183.00 ± 4.496 evening 184.00 ± 3.231.

**Fig. 8:**
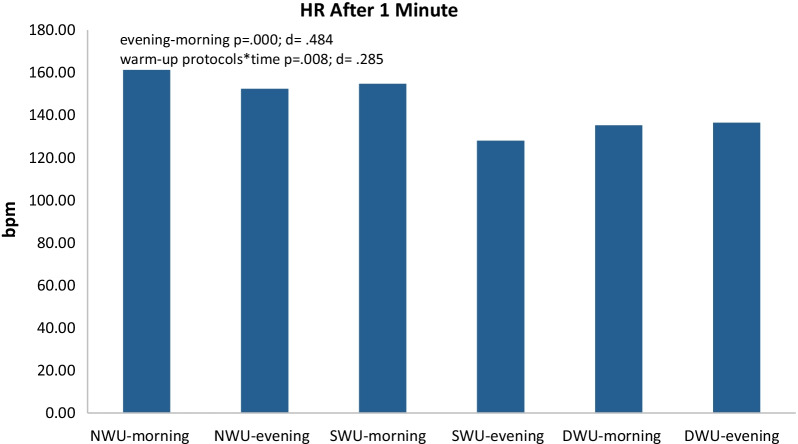
Changes after 1 min heart rate (HR) after different three warm-up protocols [no warm-up (NWU), specific warm-up (SWU), and dynamic warm-up (DWU)] in the morning and evening hours

In Fig. 8, there was a significant decrease in HR after 1 min, evening compared to the morning without discriminating three warm-up protocols (F_(1.37)=_16.91 *p* ≤ 0.001, η_p_^2^: 0.48). Moreover, warm-up protocols × time (evening and morning) interaction have an impact on SJFT for HR after 1 min (F_(1.370)=_ 7.16, *p* = 0.008, η_p_^2^: 0.29). When significant differences between groups are considered, significant difference was found between NW and SWU (*p* < 0.015) and NW and DWU (*p* ≤ 0.001). There was no significant difference in values between SWU and DWU (*p* > 0.160). When analyzed changes after 1 min heart rate values within themselves, it was determined that NWU morning 161.20 ± 8.966, evening 152.30 ± 18.809; SWU morning 154.70 ± 13.416 evening 128.00 ± 20.132; DWU morning 135.20 ± 8.816 evening 136.40 ± 8.884. Results further showed that the DWU had lower heart rate values than NWU (*p* < 0.05).

## Discussion

The aim of this study was to analyse the differences in SJFT performance assested in two different time of day (morning and evening) after three warm-ups protocols (NWU, DWU, SWU). The major finding of the present study revealed that the significant increase in SJFT performance in the evening compared to the morning and also significant linear decreases in heart rate were observed after NWU, DWU and SWU in the both morning and evening hours. Performance time can be an important factor in sports, it is essential to know the most productive working time of our body and mind in order to plan training and practice [41].

In this study, there was a significant increase in SJFT for series A, series B, series C, total number of throws and there was a significant decrease in SJFT index, after the end of all SJFT HR and one minute after the end of all SJFT in the evening compared to the morning without discriminating three warm-up protocols. There are limited circadian rhythm studies about judokas [27, 42]. But there are no studies find about circadian rhythm that assesses the SJFT performance of woman judokas. There is only one study about circadian rhythm that assesses the SJFT performance in the literature. Miarka et al. [15] examined acute effects and postactivation potentiation in the SJFT. They found that contrast and plyometric exercises performed before the SJFT can result in improvements in the test index and anaerobic power of judo athletes, respectively. about circadian rhythm that assesses the SJFT performance [15].

However, there are studies determining the effect of circadian rhythm on different performance values of judokas. Chtourou et al. reported that the repeated sprint running performance and mood of the elite athletes tested did not show a strong dependence on the time of day of the test. They stated that the reason for this result may be the habit of exercising in the early hours of the morning [43]. Chtourou et al. investigated the effect of time of day on short-term maximum performances before and after a judo match in young judokas. The results of the study reported that the muscle strength and power of judokas were significantly higher in the afternoon than in the morning. However, these diurnal variations disappeared in the afternoon after the judo competition, with more fatigue than in the morning [27]. The increase in body temperature due to diurnal variation can reflect passive muscle warm up and cause an increase in metabolic reaction, an increase in the extensibility of connective tissue, a decrease in muscle viscosity, and an increase in the rate of conduction of action potentials [44, 45]. Also, diurnal variation in body temperature may result in better motor coordination, which can produce higher peak performance in the afternoon rather than in the morning [46]. To explain the diurnal variation in performance, these diurnal improvements in muscle performance have been shown to result from improved muscle contraction properties rather than a change in neural drive modification in the evening [47, 48]. In other studies involving circadian rhythm, long and short-term exercise performance, mood, [49] lactic acid values, heart rate, anaerobic power [50] increased in the afternoon and evening hours compared to morning hours.

It may be important to include specific and dynamic warm-ups in sports performance. These exercises serve to increase the activation of performance-limiting muscles that are directly related to sports [51]. By stimulating the nerve processes, the muscles are toned and there is an increase in the state of tension [52]. Increased muscle work reduces elastic and viscous resistances in the muscle through warm up [51]. There was significant linear decreases in heart rate were observed 1 min after NWU, DWU and SWU in the both morning and evening hours. When heart rate was taken as a criterion for recovery, significant decreases was observed in the heart rate of the judo athletes after SJFT performed both in the morning and evening hours. This shows us that they have a good recovery condition. There are limited resources in the literature including judo-specific warm-up and dynamic warm up protocols on sports performance [32]. In studies examining circadian rhythm and warm up protocols, evening performances were better from morning performance on 16.1 km cycling with a 25 min warm-up protocol [53], lower extremity strength with a 5 min warm-up protocol [54], agility [55], the 505 change of direction, 10 m sprint, and change of direction deficit test [56], swim performance [57]. Besides, Souissi et al. suggested that longer warm-up protocols were recommended in the morning hours to minimize the diurnal fluctuations of anaerobic performances [58]. Previous studies reported that the upper and lower body warm up protocols before Judo Specific Fitness Test increased performance [16], and also static stretching improved the flexibility, and static stretching after dynamic warm up increased the leg force [18]. Hammerel reported that static stretching significantly decreased SJFT index, and did not affect heart rate, and throw with technique performance [32]. The reason for the increased flexibility in static stretching may be reflex inhibition. An increased strain tolerance, decreased viscoelasticity, and to some extent a reduction in muscle-external stiffness can contribute to a sustained increase in elastic range of motion [59, 60]. The reason for the improvement of leg strength after static stretching and dynamic warm-up protocols can be explained by the sequential movement of the limbs, similar to the reciprocal inhibition sequences [61, 62]. Therefore, for range of motion to continue to proliferate after dynamic stretching, reciprocal inhibition must persist for a long time after stretching, contributing to viscous and morphological changes [63].

Besides physical performance is predicted to change over the course of a menstrual cycle (MC) due to a variety of mechanisms including altered muscle activation, substrate metabolism, thermoregulation, and body composition. Female sex hormone levels may be a factor in the altered force production. This condition has the potential to impair muscle strength and power [64, 65]. Progesterone deficiency during the follicular phase is conjectured to result in increased strength and power, especially when estrogen levels peak in the late follicular phase. Additionally, it is predicted that when progesterone levels are elevated during the luteal phase, lower power results will be obtained. The MC stage can have a remarkable effect on the generation of rapid force. Muscle activation, more specifically the rate at which initial motor units fire, is the primary determinant of the rapid force generation required for explosive movements [66]. According to Shahlina et al. [67], the average number of beats completed during the menstrual phase is less than the average number of beats completed during the postmenstrual phase (grades C and A, respectively) (27.7 vs. 30.0 beats). The HR immediately following launch efforts was comparable (grade C), but the HR one minute after SJFT was significantly different (grade B vs. C). Additionally, the SJFT index varied according to menstrual and postmenstrual phases (grade C and grade B). Premenstrual and postmenstrual phases exhibit similar patterns. The SJFT index reached its maximum value (10.1) during the menstrual cycle's postovulatory phase (grade A) [67]. Štefanovský et al. [68] was to verify the effect of selected phases of the menstrual cycle on the anaerobic performance of judokas in the Wingate test and the Special judo fitness test. They discovered that, with the exception of the number of shots fired during the first 15 s of the Special judo fitness test, no significant changes in any of these parameters were observed as a result of menstrual phase changes in the Wingate and Special judo fitness tests during the luteal phase [68]. Although there is currently no consensus regarding the effect of monthly hormonal fluctuations on female performance [69], recommendations have been made indicating that regularly menstruating female athletes participating in strength-specific sports do not require menstrual cycle adjustment to maximize their competitive abilities [70]. However, possible changes in plasma volume during the menstrual cycle may have an effect on heart rate, which may need to be adjusted to maintain cardiac output [70], and on the SJFT index calculation.

As with other combat sports, judo has weight classifications. Athletes are weighed prior to each tournament to determine the weight categories in which they will compete. The weight control procedure was established to ensure that all individuals with similar characteristics had an equal opportunity to compete [71]. Athletes with similar anthropometric characteristics should theoretically have comparable physical abilities and thus be eligible to compete in the same weight category. Numerous judoka employ the well-known rapid weight loss (RWL) strategy prior to competition in order to gain an advantage over their opponents. This behavior pattern appears to be widespread among judoka [72]. Koral and Dosseville [73] conducted research to determine the effects of a combination of gradual and rapid body mass loss on the physical performance and psychological state of elite judo athletes. The results of this study indicated that when compared to four weeks prior to the championship, the experimental group demonstrated a significant decrease in body mass, estimated body fat, and judo movement repetitions over 30 s, as well as an increase in confusion and tension scores, but a decrease in vigour. There was no discernible difference in squat jump or countermovement jump performance, or in judo movement repetitions lasting longer than 5 s [73]. Morales et al [74] demonstrated negative effects on perceptual motor-skill performance in judo athletes engaging in RWL strategies prior to competition. Given the detrimental effects of RWL as documented in the current literature, it is critical to establish and monitor an athlete’s minimum competitive weight in order to prioritize the athlete’s health and safety, to emphasize fairness, and to ultimately benefit the sport [75].

In addition, when the literature is examined, there are studies examining the effect of warm up on sports performance in other combat sports (MMA, wrestling, muay thai, kickboxing) [21, 76–78]. Herman and Smith [78] were to determine whether a dynamic-stretching warm-up (DWU) intervention performed daily over 4 weeks positively influenced power, speed, agility, endurance, flexibility, and strength performance measures in collegiate wrestlers when compared to a static-stretching warm-up (SWU) intervention. Their measures included peak torque of the quadriceps and hamstrings, medicine ball underhand throw, 300-yd shuttle, pull-ups, push-ups, sit-ups, broad jump, 600 m run, sit-and-reach test, and trunk extension test. Wrestlers completing the 4 week DWU intervention had several performance improvements, including increases in quadriceps peak torque, broad jump, underhand medicine ball throw, sit-ups, and push-ups. A decrease in the average time to completion of the 300-yd shuttle and the 600 m run was suggestive of enhanced muscular strength, endurance, agility, and anaerobic capacity in the DWU group [78]. Bayer and Özgür [76], were to evaluate the acute effect of different massage times on squat jump, countermovement jump and flexibility performance. There was find a significant main effect for flexibility, countermovement jump and squat jump performance of muay thai athletes [76]. Eken and Bayer [77], had evaluated the effects of proprioceptive neuromuscular facilitation (PNF) stretching, massage, PNF + massage on flexibility, vertical jump and hand grip strength performance in kickboxers. They found that, there was a significant difference between PNF and PNF + M, M and PNF + M in favour of PNF + M in vertical jump. They found a significant decrease in right- and left-hand grip strength for all protocols [77].

## Conclusion

In conclusion, the present study confirms that time-of-day and warm-up protocols (not significant except HR after warm up protocols and index) have significant effects on SJFT performances. SWU practice is a warm-up that imitates judo techniques, and is associated with the characteristic structure of judo. Increased body temperature with SWU in the evening may have triggered a further increase in SJFT performance. There was significant linear decreases in HR were observed in SJFT after 1 min on NWU, DWU and SWU in the both morning and evening hours. In sum, the results of the present study suggest that SWU protocol is sufficient to alter SJFT performance in the evening hours. This can be taken into account when planning training programs. This study includes some limitations. Afternoon hours were not evaluated in this study, and also menstrual cycle periods of women athletes were not taken into account. The study can be repeated by increasing the sample size in men and women elite and top elite judo players of different age groups. Increasing the number of studies examining the effects of different interval exercise protocols, warm up protocols, stretching protocols and circadian rhythm on different performance parameters in Judo (Uchikomi Fitness Test, Judo Specific Fitness Test, Santos Test etc.) may give more some specific recommendations about the planning of judo-specific warm-up exercises before training programs. However, it is conceivable that improvement of test performances could be achieved with a warm-up for elevating HR and muscle temperature with RAMP protocols. This can contribute to judo athletes getting maximum efficiency from their judo performance both before training and competitions, and minimizing the risk of injury.

## Supplementary Information

**Additional file 1.** All article information can be viewed in this section.

## Data Availability

The datasets generated and analysed during the current study are publicly available in the following link: https://dataverse.harvard.edu/dataset.xhtml?persistentId=doi:10.7910/DVN/MZT1PW.

## Abbreviations

SJFT: Special judo fitness test
Hrrest: Resting heart rate
NWU: No warm up
SWU: Specific warm up
DWU: Dynamic warm up
